# For severe and rigid adult idiopathic scoliosis, does an optimal extent of posterior intervertebral release exist? A finite element analysis

**DOI:** 10.3389/fbioe.2025.1691245

**Published:** 2025-11-24

**Authors:** Zhen Chen, Jianqun Zhang, Xiaoyin Liu, Rong Ma, Simin Liang, Zhaohui Ge

**Affiliations:** 1 Department of Orthopedic, General Hospital of Ningxia Medical University, Yinchuan, China; 2 First Clinical Medical College of Ningxia Medical University, Yinchuan, China

**Keywords:** adult idiopathic scoliosis, finite element analysis, posterior intervertebral release, ponte osteotomy, optimal release

## Abstract

**Background:**

The surgical management of severe, rigid adult idiopathic scoliosis (AdIS) is challenging. While posterior intervertebral release (PIVR) is used to enhance correction, the optimal number of release segments is unknown. This study aimed to identify the optimal PIVR strategy by evaluating the biomechanical effects of varying release levels.

**Methods:**

A patient-specific finite element (FE) model of a Lenke 2A + AdIS spine (T1-sacrum, main thoracic curve 84°) was created and validated. Six surgical scenarios were simulated: instrumentation-only (M1), Ponte osteotomy alone (M2), and M2 combined with four different PIVR strategies (M3-M6). Simulated corrective forces were applied, and outcomes, including Cobb angle correction and von-Mises stress on vertebrae and implants, were analyzed and compared.

**Results:**

A targeted 4-level PIVR centered on the apex (M5) achieved the greatest main thoracic curve correction, reducing the Cobb angle from 84° to 34.88° (a 58.5% correction rate). A more extensive 6-level release (M6) proved less effective (38.99°, 53.6% correction rate). Model M5 also produced the most significant reduction in peak von-Mises stress on vertebrae (15.9% decrease vs. M2) and pedicle screws (32.9% decrease vs. M2).

**Conclusion:**

A selective, 4-level PIVR strategy provides superior deformity correction and a more favorable stress environment than a more extensive release. These findings challenge the “more is better” paradigm, providing a biomechanical rationale for an “optimal” rather than “maximal” release approach in severe rigid AdIS.

## 1 Introduction

The surgical management of severe, rigid adult idiopathic scoliosis (AdIS), defined by a Cobb angle >70° and flexibility <30%, presents a formidable clinical challenge (Teixeira da Silva et al., 2015; Kandwal et al., 2017). While three-column osteotomies (3COs) like vertebral column resection (VCR) offer powerful correction, they carry high rates of major complications (Auerbach et al., 2012; Riley et al., 2018; Van Halm-Lutterodt et al., 2023). Consequently, surgeons often prefer a posterior-only approach combining pedicle screw instrumentation with less aggressive releases like the Ponte osteotomy (Ponte et al., 2018; Faldini et al., 2021). However, for the most rigid curves, the corrective power of Ponte osteotomy alone is often insufficient (Huang et al., 2021), necessitating the development of more effective yet less invasive surgical strategies.

To address this unmet clinical need, posterior intervertebral release (PIVR) has been introduced as a promising supplemental technique. PIVR, performed through a posterior approach, involves a complete discectomy with resection of the anterior and posterior longitudinal ligaments, as well as a portion of the vertebral endplates. This extensive release of the anterior and middle spinal columns is biomechanically designed to significantly reduce spinal rigidity and increase segmental mobility (Zhu et al., 2023; Deng et al., 2024), thereby creating a more favorable mechanical environment for correction.

Despite its theoretical advantages, a critical bioengineering knowledge gap remains. The quantitative biomechanical benefit of PIVR, particularly the non-linear relationship between the number of released segments and the resulting gains in spinal flexibility and correction, is poorly understood, which prevents surgeons from optimizing this technique in a data-driven manner, potentially leading to suboptimal outcomes or elevated risks of implant-related complications. Therefore, this study employs a computational biomechanics approach using finite element analysis (FEA) to systematically investigate this dose-response relationship, with the goal of identifying the optimal PIVR strategy that maximizes correction while minimizing implant stress. We hypothesized that an optimal, rather than maximal, number of releases would yield the most favorable biomechanical results.

## 2 Materials and methods

### 2.1 Construction and meshing of the intact FE model

A 24-year-old male patient with AdIS (176 cm, 67 kg) was included. This patient presented with a main thoracic (MT) curve of 84° and a proximal thoracic (PT) curve of 48°. On supine bending radiographs, the MT and PT curves corrected to 72° and 29°, respectively, yielding a flexibility index of 14.3% for the MT curve, which meets the criteria for severe and rigid scoliosis. The study protocol was approved by the Institutional Ethics Committee (Approval No. 2022-1137), and informed consent was obtained from the participant.

A high-resolution computed tomography (CT) scan (0.625 mm slice thickness) of the T1-sacrum was acquired. The DICOM images were imported into Mimics Medical 21.0 (Materialise, Belgium) to generate a preliminary 3D model of the T1-pelvis via threshold segmentation and manual editing. This model was then processed in Geomagic Wrap 2021 (3D Systems, United States) for smoothing, denoising, and reverse engineering to create a solid geometric model of the T1-sacral vertebrae. The vertebral models were assembled in Solidworks 2018 (Dassault Systèmes, France). Intervertebral discs (IVDs) and facet joint cartilage were reconstructed based on anatomical relationships. The IVD was composed of a nucleus pulposus (44% by volume) and an annulus fibrosus (56% by volume) (Zhong et al., 2023; Wang et al., 2025). The endplates and facet cartilage were assigned a uniform thickness of 1.0 mm and 0.2 mm, respectively. This resulted in the intact T1-sacrum AdIS model, designated as M0.

**FIGURE 1:**
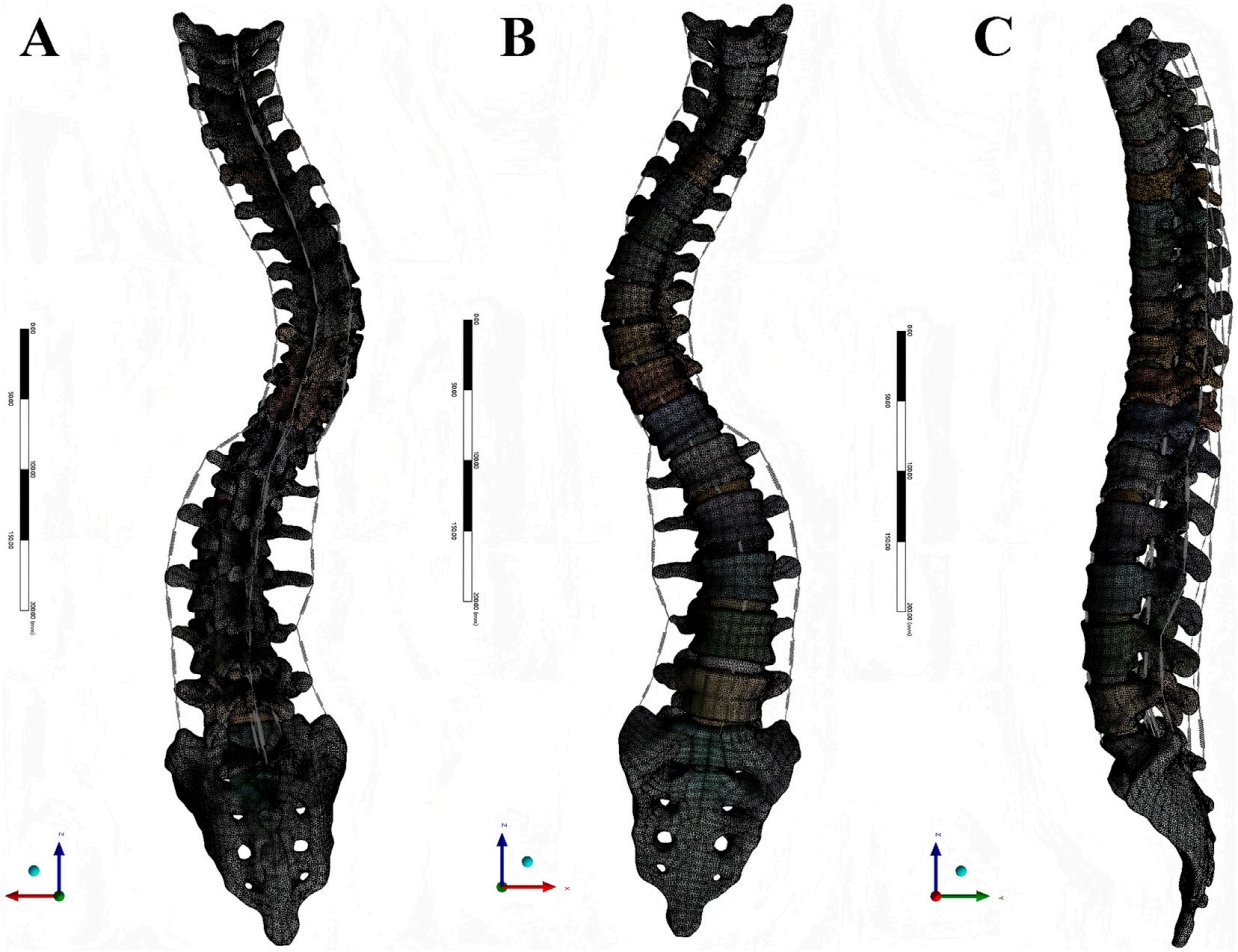
The intact T1-sacrum finite element model. **(A)** Posterior view. **(B)** Anterior view. **(C)** Lateral view.

The final model was imported into ANSYS Workbench 18.0 (ANSYS, Inc., United States) for preprocessing. A mesh convergence study was performed to ensure that the results were independent of mesh density. The final mesh for the intact model consisted of 1,657,019 elements and 375,436 nodes (Figure 1). The main spinal ligaments (anterior and posterior longitudinal, ligamentum flavum, interspinous, supraspinous, and intertransverse) were simulated using nonlinear spring elements based on anatomical attachment points. The material properties of all components, derived from established literature (Zhou et al., 2020; Yang et al., 2023; Wang et al., 2024), are detailed in Table 1.

**TABLE 1:** Material properties of the T1-sacrum finite element model.

Components	Young’s modulus (MPa)	Poisson’s ratio	Stiffness coefficient (N/mm)	References
Cortical bone	12,000	0.3	—	Bereczki et al. (2021) Wang et al. (2021)
Cancellous bone	100	0.2	—	Bereczki et al. (2021) Wang et al. (2021)
Endplate	2000	0.2	—	Wang et al. (2021) Fan et al. (2023)
Anulus fibrosus	4.2	0.45	—	Wang et al. (2021) Fan et al. (2023)
Nucleus pulposus	1	0.499	—	Wang et al. (2021) Fan et al. (2023)
Articular cartilage	25	0.25	—	Wang et al. (2021) Fan et al. (2023)
Ligaments				Kumaran et al. (2021)
Anterior longitudinal ligament	7.8	—	8.74	
Posterior longitudinal ligament	10	—	5.83	
Supraspinous ligament	8	—	15.38	
Interspinous ligament	10	—	0.19	
Ligamentum flavum	15	—	15.75	
Intertransverse ligament	10	—	2.39	
Internal fixation (Ti-6A1-4V)	11,000	0.28	—	Bereczki et al. (2021)

### 2.2 Validation of the intact FE model

The intact T1-sacrum FE model (M0) was rigorously validated by replicating the loading and boundary conditions of established *in vitro* experiments and comparing the predicted biomechanical responses.

**FIGURE 2:**
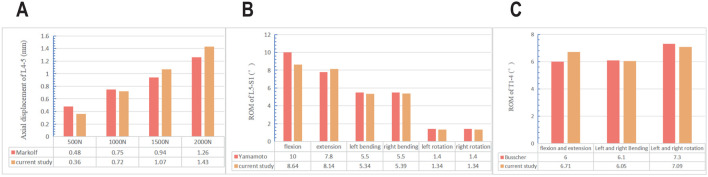
Comparison of the range of motion and axial displacements between the intact model and *in vitro* studies **(A)** axial displacements of L4-5; **(B)** range of motion of L5/S1; **(C)** range of motion of T1/4.

Cervicothoracic Junction Validation: The model was validated against the *in vitro* study by Busscher et al. (2009). To precisely replicate their experimental setup, a pure moment of 4.0 N m was applied to the superior surface of the T1 vertebral body, while the inferior surface of T4 was fully fixed in all degrees of freedom. The computed range of motion (ROM) of the T1-T4 segment in flexion, extension, lateral bending, and axial rotation was found to be within one standard deviation of the mean values reported in the experimental data (Figure 2C).

Lumbosacral Junction Validation: The validation of the lumbar segments was conducted in two steps: ROM Validation: Following the protocol of Yamamoto et al. (1989), a pure moment of 10.0 N m was applied to the superior surface of L5 with the sacrum fully fixed. The resulting ROM of the L5-S1 segment in all primary planes of motion showed excellent agreement with the experimental results (Figure 2B).

Axial Compression Validation: The axial stiffness of the L4-5 segment was validated against the work of Markolf. (1972). A compressive load from 500 N to 2000 N was applied to the superior surface of L4, while the inferior surface of L5 was fixed. The resulting axial displacement closely matched the experimental data (Figure 2A).

While direct *in vitro* data for severely rigid scoliotic thoracic segments is scarce, preventing a direct validation of the primary deformity region (T5-T12), the model’s demonstrated fidelity in the adjacent mobile segments, combined with its high-resolution, patient-specific geometry, provides a robust and reliable basis for the comparative analysis conducted between the different surgical simulation models in this study.

### 2.3 Surgical simulation and model groups

Based on the validated intact model (M0), six surgical models were created to simulate different corrective strategies. The Lenke 2A + curve was instrumented from the upper instrumented vertebra (UIV) at T2 to the lower instrumented vertebra (LIV) at L2 using a 5.5 mm titanium alloy (Ti6Al4V) pedicle screw and rod system (UPASS-2, WEGO, China). A critical-vertebra screw placement strategy was employed.

**FIGURE 3:**
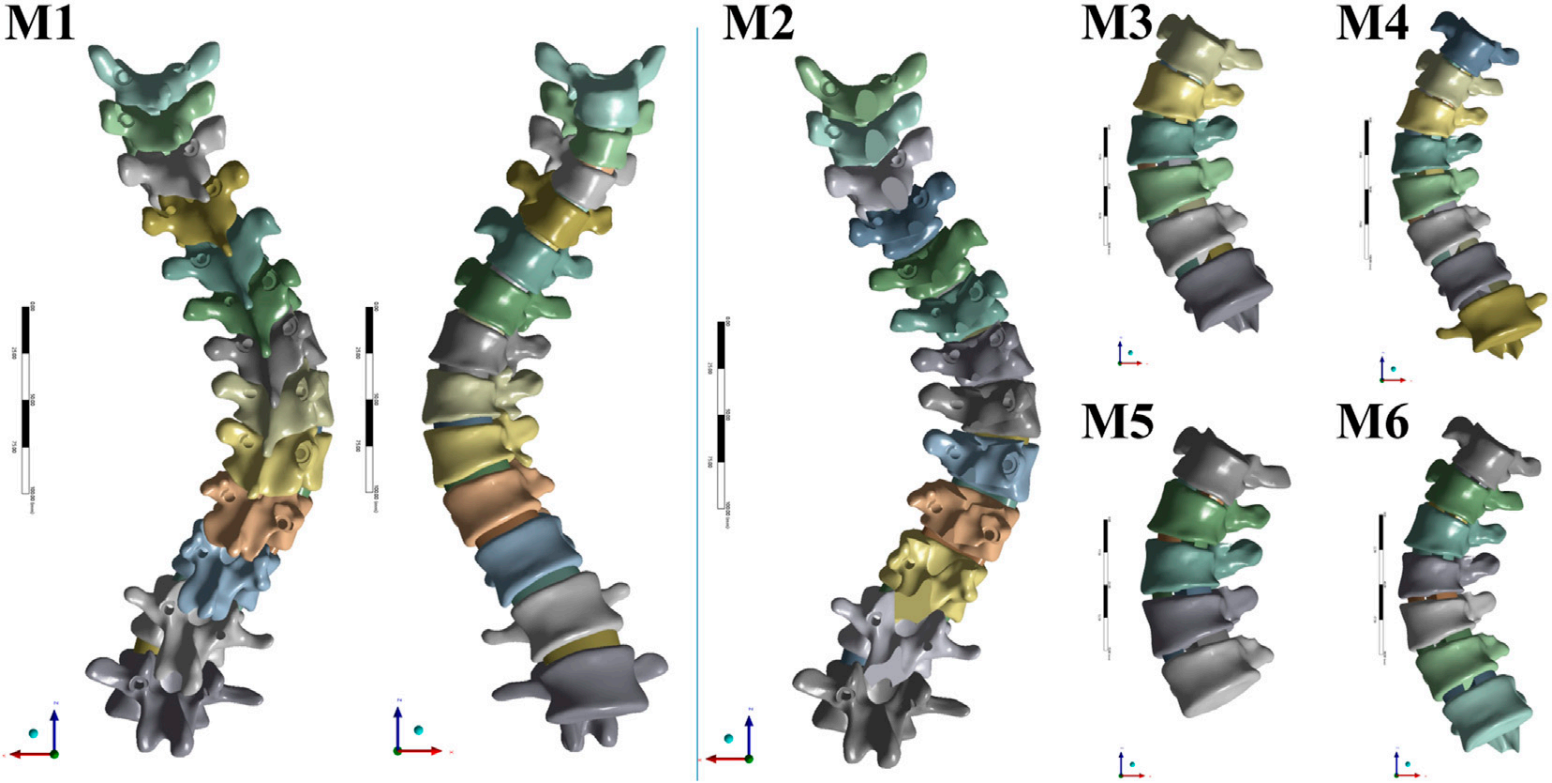
Six models including instrumentations, Ponte osteotomy and posterior intervertebral release (PIVR).

The surgical simulations were defined as follows (summarized in Figure 3):

M1 (Instrumentation-Only): Served as the baseline control, with only pedicle screw instrumentation applied from T2 to L2.

**FIGURE 4:**
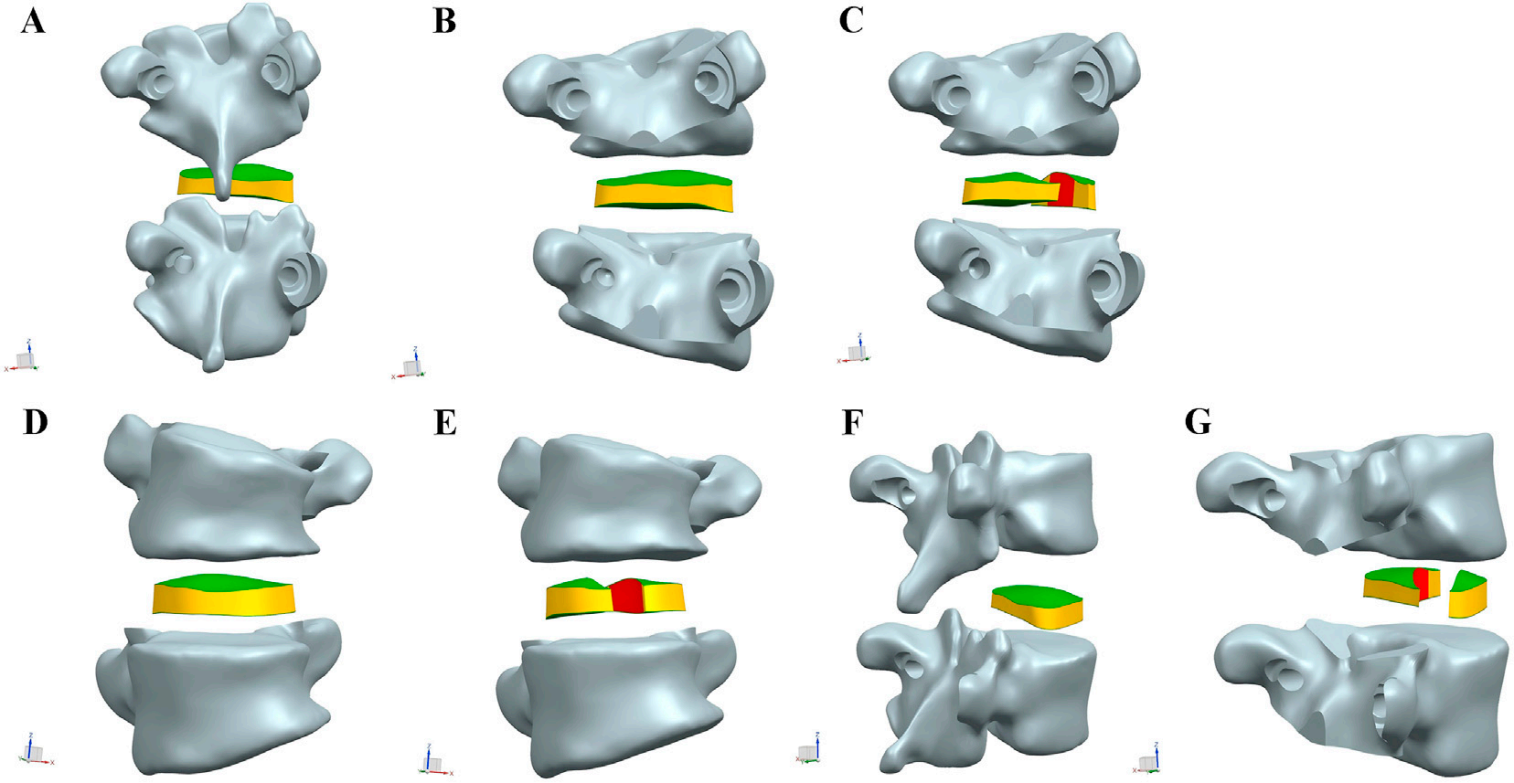
Exploded view of the Ponte osteotomy and posterior intervertebral release (PIVR). **(A–C)** Views from the posterior perspective. **(D–E)** Views from the anterior perspective. **(F–G)** Views from the lateral perspective.

M2 (Ponte Osteotomy): In addition to M1 instrumentation, a multi-level Ponte osteotomy was simulated from T5 to T12. This was modeled by the complete removal of the spinous processes, laminae, ligamentum flavum, and facet joints within these segments (Figure 4).

**FIGURE 5:**
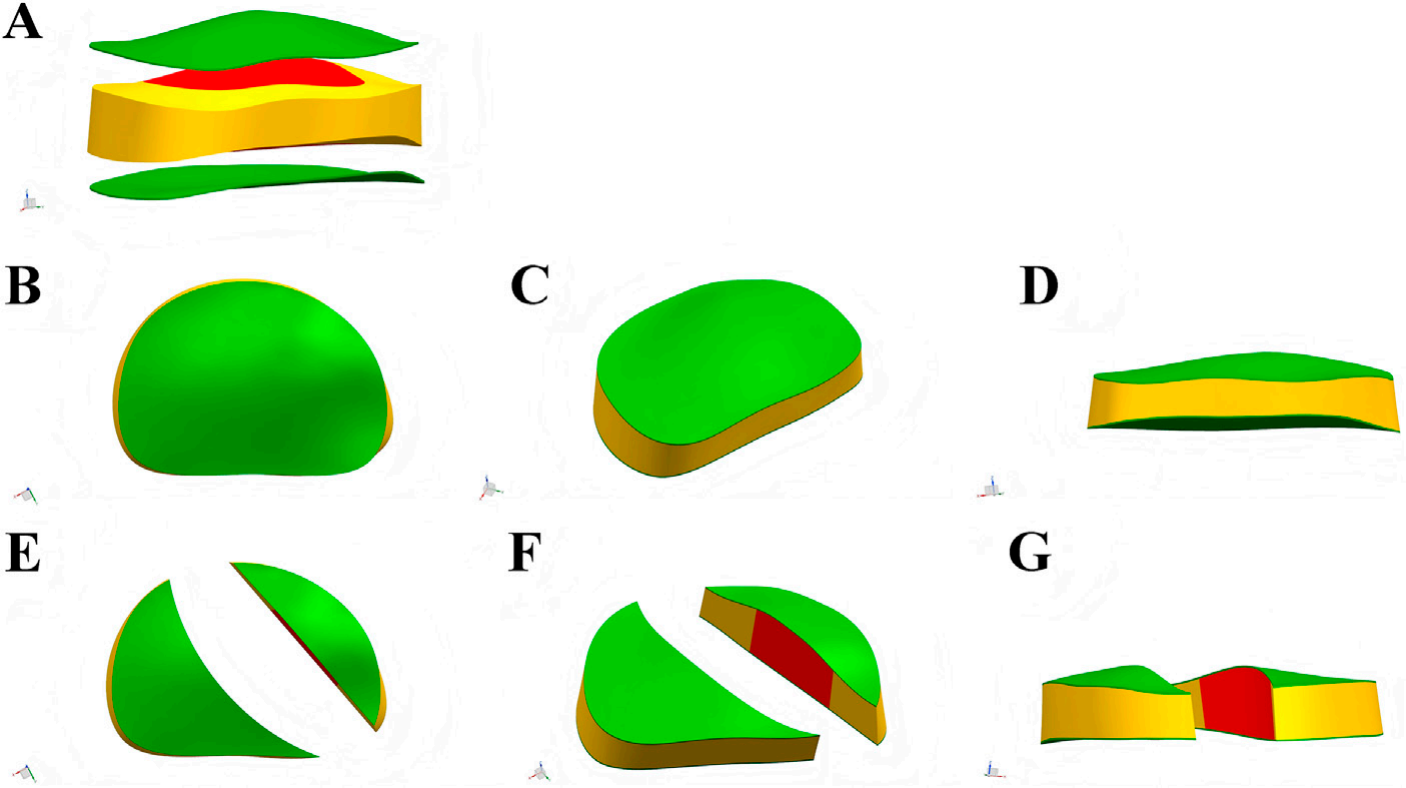
Multi-perspective comparison of intervertebral disc models before and after posterior intervertebral release (PIVR). **(A)** Schematic diagram of the original intervertebral disc model. **(B–D)** Views of the model before PIVR. **(E–G)** Views of the model after PIVR.

M3-M6 (Ponte + PIVR):

These models evaluated additional PIVR effects through convex-side transforaminal approach with 30% posterior disc resection (Figures 4, 5). The designs followed two distinct surgical philosophies:

Anatomical Region-Based Release:

M3: PIVR across the entire main thoracic curve (T7-T12, 5 levels).

M4: Extended release including transitional zones (T6-L1, 7 levels).

Apical-Targeted Release:

M5: Focused release around apical vertebra T9 ±2 levels (T7-T11, 4 levels).

M6: Extended apical release T9 ±3 levels (T6-T12, 6 levels).

### 2.4 Loading and boundary conditions

In all surgical models, the sacrum was fully constrained in all six degrees of freedom. To simulate the primary translational component of the surgical correction maneuver, a series of lateral forces (along the X-axis) were applied to the pedicle screw heads on the convex side (right side). Basis for Load Determination: The magnitude of the lateral loads was determined based on the typical stress range that pedicle screws may experience during the correction process. As shown in Table 2, the stress values of the screws ranged from 150 MPa to 394 MPa. This range is consistent with the intraoperative screw stress levels reported in the literature (Cho et al., 2010; Thiele et al., 2007), ensuring the clinical relevance of the loads. Contact Condition Definitions: The contact between the screws and bone was defined as bonded to simulate the ideal state of osseointegration. The contact between the rods and screws was also defined as bonded to simulate the final locked state of the internal fixation system.

**TABLE 2:** Loading of screw force in T4-L1 along the X-axis (N).

Vertebrae	Pedicle screw(L)	Pedicle screw(R)
T4	—	118
T5	187	187
T6	369	—
T7	150	150
T8	180	180
T9	200	200
T10	180	180
T11	150	150
T12	394	—
L1	245	245

### 2.5 Measurement of coronal Cobb angles

The coronal Cobb angles for both the main thoracic (MT) and proximal thoracic (PT) curves were measured digitally on the finite element model using a standardized protocol to ensure consistency and reproducibility. The measurement procedure was performed within the ANSYS Workbench post-processing module, adhering to the standard clinical principle: the Cobb angle is defined as the angle between the superior endplate of the most cephalad vertebra and the inferior endplate of the most caudad vertebra within the curve that are maximally tilted toward each other. The specific measurement procedure was conducted as follows: Definition of the Coronal Plane: A best-fit coronal plane was defined for the entire spinal model to ensure all angular measurements were performed within a consistent anatomical coordinate system. Identification of End Vertebrae and Nodes: The upper instrumented vertebra (UIV) and lower instrumented vertebra (LIV) defining the MT (T5-T12) and PT (T1-T4) curves were identified. The spatial coordinates of all nodes on the superior endplate of the UIV and the inferior endplate of the LIV were then extracted. Calculation of Endplate Normal Vectors: The extracted nodal coordinates were used to perform a least-squares fit to determine the geometric plane of each endplate. The unit normal vectors to these fitted planes, denoted as n_UIV (superior endplate) and n_LIV (inferior endplate), were calculated. Calculation of the Cobb Angle: The Cobb angle was derived from the angle (θ) between the two unit normal vectors, n_UIV and n_LIV, using the standard vector dot product formula. The Cobb angle is the supplement of this angle. The formulas used for the calculation are:

$$\theta =\arccos\left(|n_{\text{UIV}}\cdot n_{\text{LIV}}|\right)$$

Cobb Angle=180°−θ

where | | denotes the absolute value, ensuring a positive angle.

## 3 Results

### 3.1 Coronal plane correction and maximum global displacement

**FIGURE 6:**
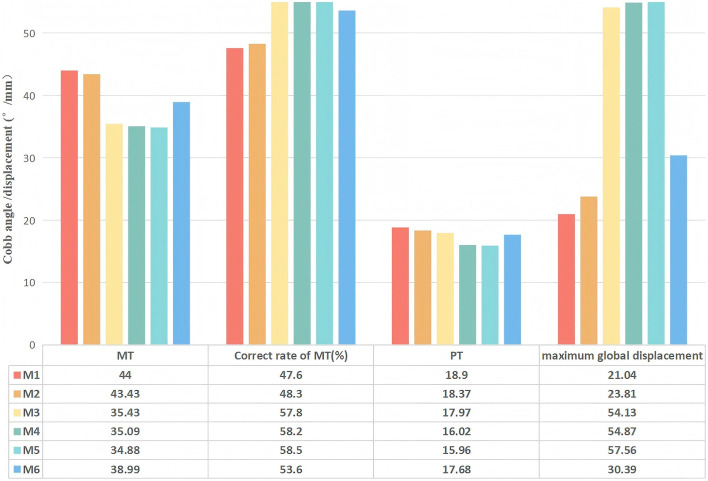
Comparison of Cobb angle of MT and PT and global displacement of M1-M6.

The postoperative coronal Cobb angles and correction rates for the MT and PT curves were presented in Figure 6.

Instrumentation-only (M1) and the addition of Ponte osteotomy (M2) resulted in modest corrections of the MT curve, reducing the Cobb angle from 84° to 44° (47.6% correction) and 43.43° (48.3% correction), respectively.

All PIVR models (M3-M6) achieved markedly superior correction of the MT curve. Notably, a non-linear relationship was observed between the number of released levels and the corrective outcome. The optimal result was achieved in M5 (apical ±2 level PIVR), which reduced the MT Cobb angle to 34.88°, achieving a correction rate of 58.5%. Counter-intuitively, the more extensive 6-level release in M6 yielded a less effective correction (38.99°, 53.6% correction rate), which was inferior to that of M5.

Changes in the PT curve were minimal and not significantly different across all surgical models.

**FIGURE 7:**
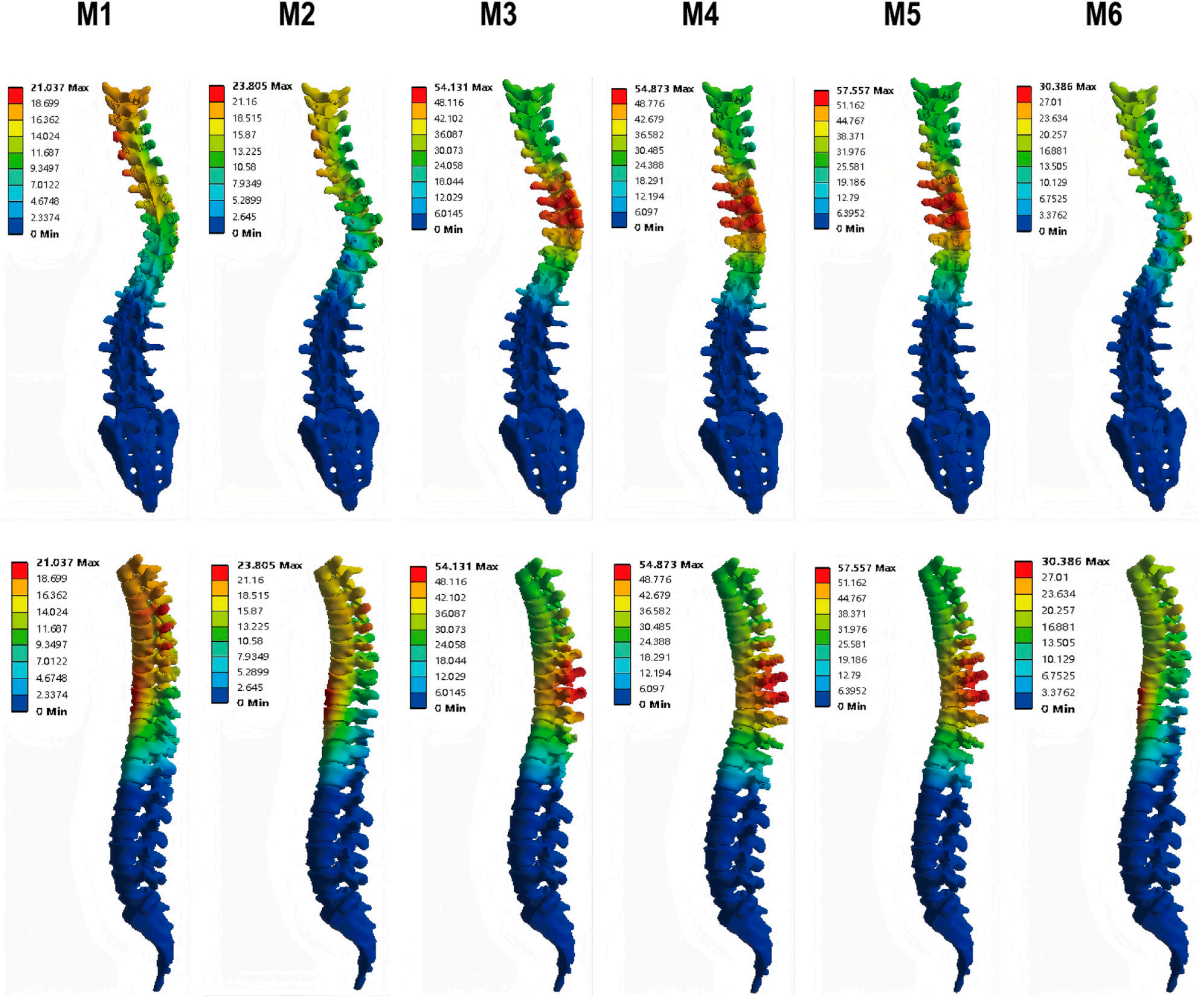
Cloud of global displacement for M1-M6.

The maximum global displacement, quantifying the overall corrective shift of the spinal column, demonstrated substantial variation among the surgical models. Relative to the instrumentation-only construct (M1, 21.04 mm), models incorporating additional releases—M3 (54.13 mm), M4 (54.87 mm), and M5 (57.56 mm)—exhibited significantly greater displacements, corresponding to increases of 157%, 161%, and 174%, respectively. Among all models, M5 yielded the peak displacement, indicating its superior efficacy in mobilizing the spine. In contrast, M2 (23.81 mm) and M6 (30.39 mm) resulted in more moderate gains (Figure 6). These quantitative findings are corroborated by the displacement distribution patterns in Figure 7, which visually affirm the most extensive and uniform correction field achieved with model M5.

### 3.2 Maximum von-mises stress on vertebrae

**FIGURE 8:**
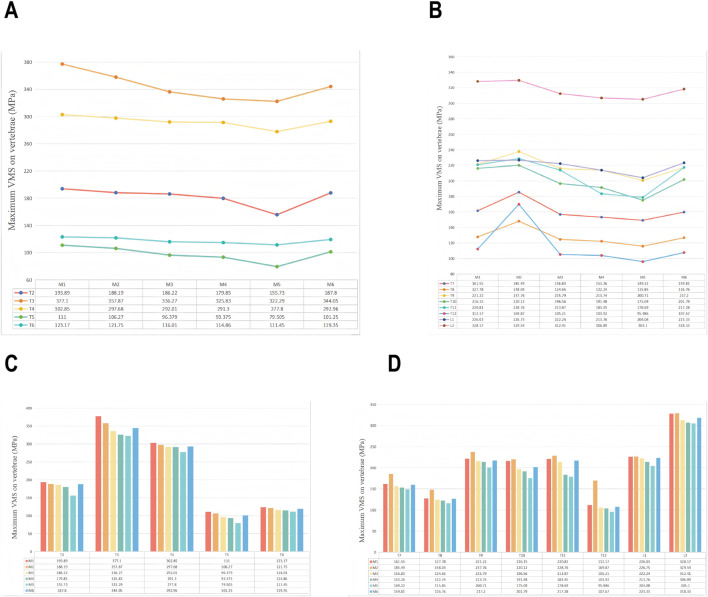
Comparison of the maximum von-Mises stress on vertebrae of PT **(A,C)** and MT **(B,D)**.

**FIGURE 9:**
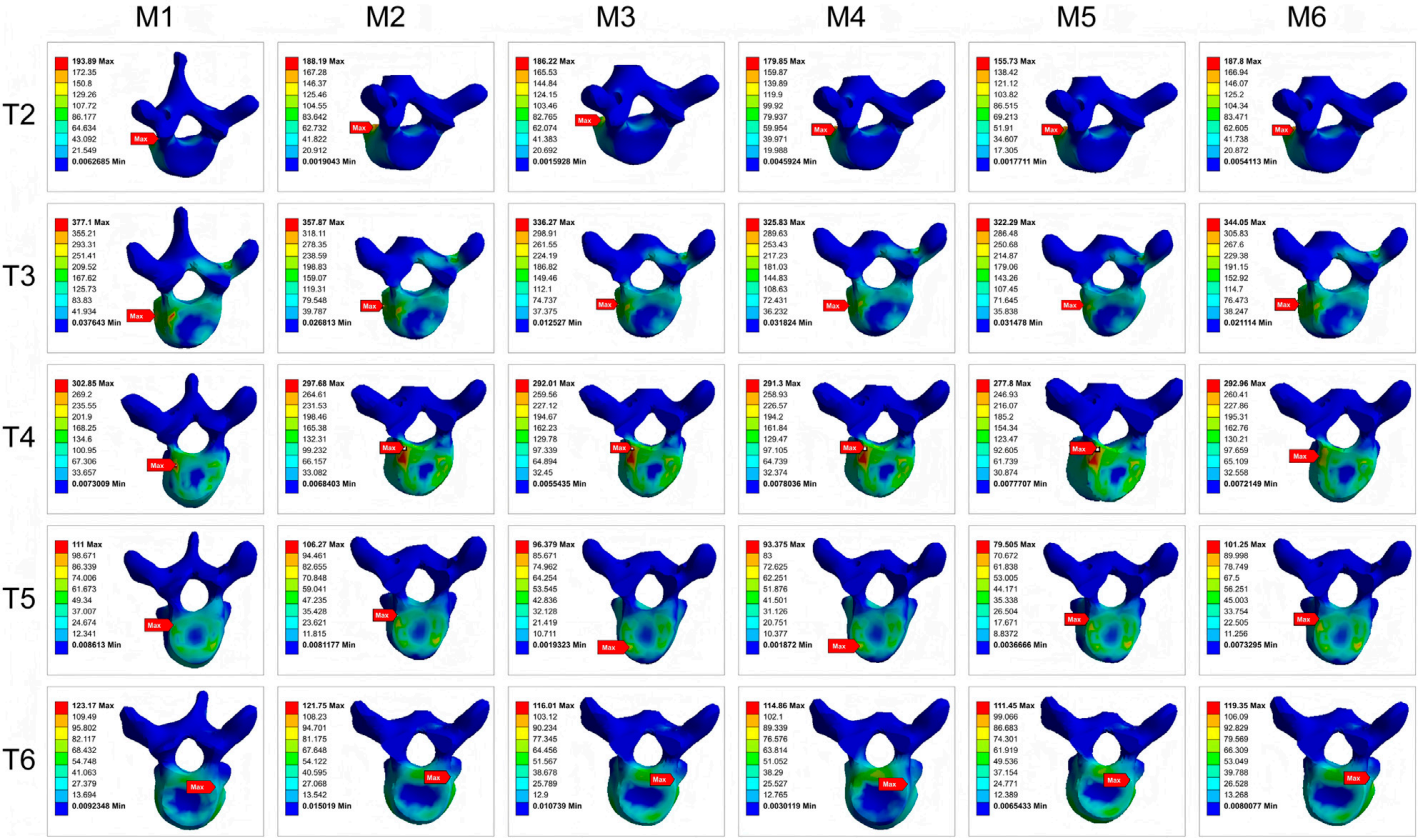
Maximum von-Mises stress clouds on PT vertebrae for models M1-M6.

**FIGURE 10:**
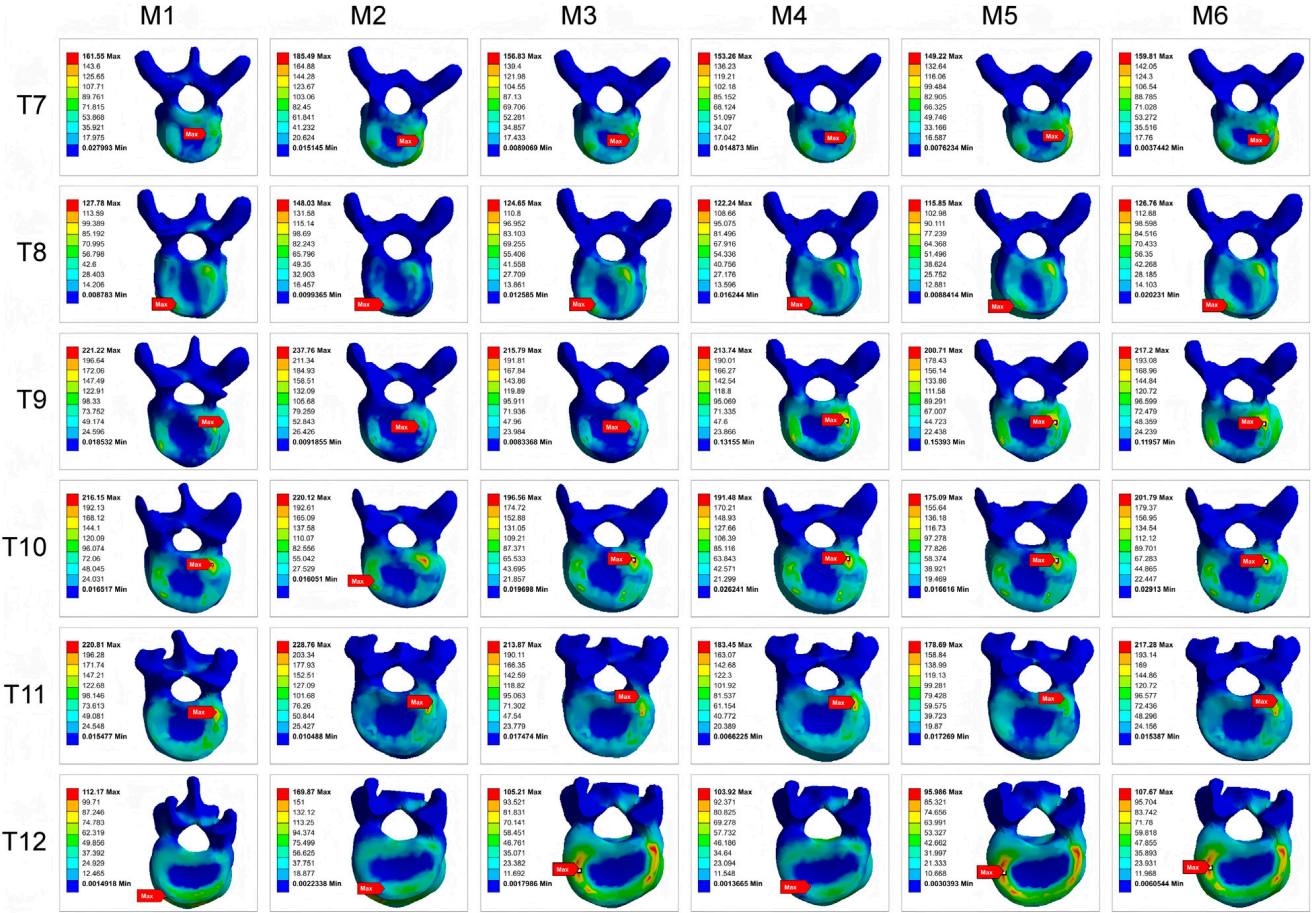
Maximum von-Mises stress clouds on MT vertebrae for models M1-M6.

The maximum von-Mises stress on the vertebrae within the instrumented segment (T2-L2) was analyzed to assess the risk of mechanical failure (Figures 8–10). The Ponte osteotomy in M2 redistributed stress, decreasing it in the flexible PT curve but increasing it in the rigid MT curve compared to M1.

All PIVR models (M3-M6) demonstrated a significant reduction in the maximum von-Mises stress on the vertebrae compared to M1 and M2, indicating that PIVR effectively relieved stress across the rigid deformity. Among the PIVR models, M5 exhibited the most favorable stress distribution, with an average maximum von-Mises stress of 182.42 MPa. This represented a 15.9% reduction in stress compared to the M2 model (216.78 MPa), suggesting the lowest risk of vertebral fracture.

### 3.3 Maximum von-mises stress on pedicle screws

**FIGURE 11:**
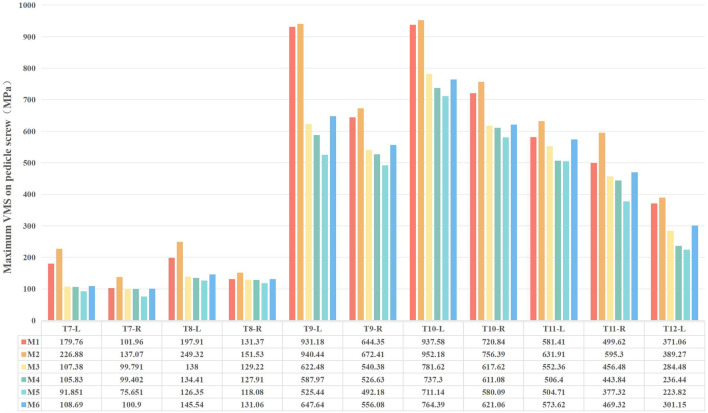
Comparison of maximum von-Mises stresses on Pedicle Screws in the MT Curvature.

**FIGURE 12:**
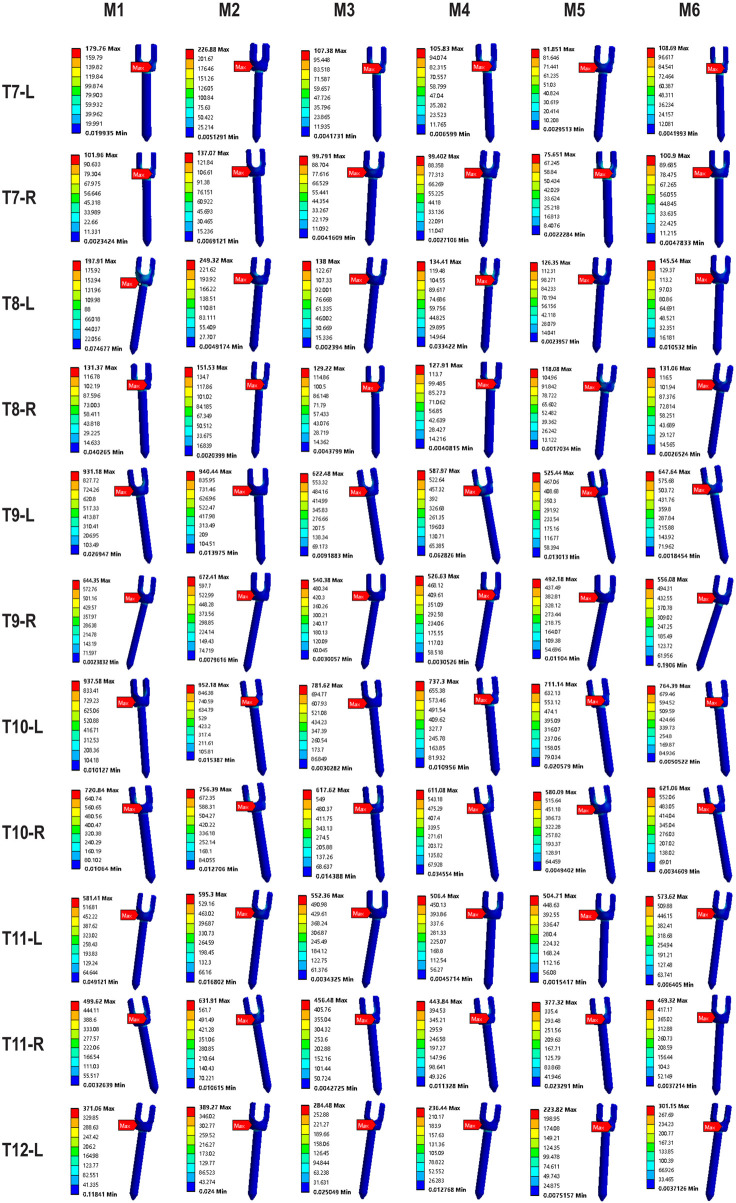
Maximum von-Mises stress clouds on MT pedicle screws of models M1-M6.

Peak von-Mises stress on the pedicle screws, an indicator of implant fatigue failure risk, was evaluated in the MT curve (Figures 11, 12). Across all models, screws on the concave side experienced higher stress than those on the convex side. The addition of Ponte osteotomy (M2) slightly increased the peak screw stress compared to M1.

Crucially, all PIVR models (M3-M6) significantly lowered the stress on the pedicle screws. The most substantial reduction was observed in M5, where the average peak stress in the screw was 347.8 MPa. This was a 32.9% decrease compared to the peak stress in M2 (518.4 MPa), indicating that the optimal release strategy not only improves correction but also creates a less demanding mechanical environment for the instrumentation. This peak stress is well below the recognized fatigue endurance limit for medical-grade titanium alloy (Ti6Al4V) under ASTM F136 standards (approx. 450 MPa), suggesting a high safety margin against long-term implant failure.

## 4 Discussion

The surgical management of severe rigid AdIS remains a formidable challenge (Teixeira da Silva et al., 2015; Kandwal et al., 2017; Ansari et al., 2024). This study, to our knowledge, is the first finite element analysis to biomechanically investigate the strategy of supplementing multi-level Ponte osteotomies with a varying number of PIVR (Petrosyan et al., 2023). Our primary findings are twofold: first, the addition of PIVR to Ponte osteotomies significantly enhances coronal plane correction and reduces stress on the vertebrae and implants compared to instrumentation alone or with Ponte osteotomies. Second, and more critically, we identified a non-linear relationship between the extent of release and corrective efficacy. A targeted, 4-level PIVR centered on the curve apex (M5) yielded the most significant correction and the most favorable stress environment, outperforming even a more extensive 6-level release (M6).

The counterintuitive finding that the M6 model produced a suboptimal correction compared to M5 warrants a detailed biomechanical explanation. This phenomenon suggests that for severe rigid deformities, maximizing the number of released segments is not synonymous with optimizing the corrective outcome. We propose two interconnected mechanisms to explain this observation. The first is the “Optimal Stiffness Fulcrum” hypothesis. In the M5 model, the preserved stiffness of the un-released segments at the ends of the instrumented construct (T6-7 and T11-12) likely acts as a stable fulcrum. This stability allows the corrective forces applied by the rod to be transmitted and concentrated efficiently at the rigid apical region (T7-T11), maximizing deformity correction. In contrast, the more extensive release in M6 created hypermobile segments at the construct’s ends. These “floppy” segments may buckle or absorb the corrective energy through excessive local motion, thereby dissipating the force rather than effectively transmitting it to the apex. This hypothesis is further strengthened by the performance of the M4 model. Although M4 involved the most extensive release (7 levels), its corrective outcome was comparable to, but not superior than, the targeted 4-level release in M5. This provides a clear illustration of the law of diminishing returns in spinal release. The additional releases in M4, particularly in the transitional zones, likely exacerbated the loss of the stable fulcrum without delivering a proportional gain in apical correction. This critical comparison between M4 and M5 clarifies the surgical trade-off: while some flexibility is necessary, there is an optimal threshold beyond which further releases merely create iatrogenic instability without enhancing the primary corrective goal. The second, related mechanism is “Corrective Force Shielding.” In M6, the corrective force is distributed over a longer, more flexible spinal segment. This wider distribution may “shield” the most rigid apical vertebrae from receiving the critical level of stress required to achieve maximal correction. M5, by focusing the release, ensures the corrective stresses are targeted precisely where they are needed most. This aligns with fundamental biomechanical principles suggesting that a balance between flexibility and stability is crucial for effective deformity correction in long-construct fusions.

The results of this study also provide a biomechanical rationale for emerging clinical reports (Mac-Thiong et al., 2016; Mikhail et al., 2020; Zhu et al., 2023). Ponte osteotomy alone (M2) showed no significant improvement in coronal correction over instrumentation-only (M1) in our rigid model. This supports the clinical observation that for severe, rigid curves, a posterior-column-only shortening procedure like Ponte osteotomy is often insufficient (Huang et al., 2021). Its value is likely maximized in more flexible curves or for sagittal plane correction (Ponte et al., 2018). The significant improvement seen in all PIVR models (M3-M6) highlights the critical role of the anterior column, specifically the intervertebral disc, in contributing to spinal rigidity. As shown in prior cadaveric studies (Wang et al., 2015; Rynearson et al., 2019), a discectomy provides a far greater increase in segmental motion in all three planes compared to a facetectomy alone. Our results quantify this effect in a patient-specific deformity model, demonstrating that releasing the anterior column via PIVR is essential for “unlocking” the rigid spine.

From a clinical perspective, these findings advocate for a strategic shift from a “maximal release” to an “optimal release” paradigm. Our data suggest that a targeted PIVR of the five segments at the apex of the deformity (apical vertebra ±2 levels) offers the best balance between achieving powerful correction and maintaining construct stability. This approach has significant practical implications. By avoiding unnecessary releases in transitional zones, surgeons may potentially reduce operative time, minimize blood loss, and lower the risk of iatrogenic instability or neurological complications associated with excessive spinal destabilization, such as nerve root traction during the convex-side approach (Zhu et al., 2023). The clinical observation that releasing transitional zones can ease rod seating might still hold true, but our study suggests this ease may come at the cost of final corrective efficacy.

Finally, the limitations of this study must be acknowledged. First, and most importantly, this study utilized a finite element model derived from a single patient with a Lenke 2A + curve. While this high-fidelity, patient-specific approach was instrumental in isolating the biomechanical effect of PIVR extent by controlling for anatomical variability, it inherently limits the generalizability of the quantitative results to other curve types (e.g., Lenke 1 or 3) or patient populations. The specific ‘optimal’ number of release levels identified here should therefore be interpreted as a proof-of-concept within this anatomical context. However, the fundamental biomechanical principles uncovered—such as the law of diminishing returns with extensive releases and the critical need for an optimal stiffness fulcrum—are likely to be widely relevant to the surgical planning of severe rigid deformities. Future studies must expand to include a larger cohort of virtual patients with diverse curve morphologies to validate and refine these findings. Second, the model did not include the rib cage or surrounding musculature, both of which contribute to the overall stiffness of the thoracic spine. Third, the material properties of the spinal components were simplified and assumed to be isotropic and homogeneous; for instance, the hyperelastic properties of the annulus fibrosus were not simulated. Fourth, our loading protocol was a simplification of the complex, multi-step surgical correction process and did not include a derotational moment. Fifth, our finite element model is fundamentally a biomechanical investigation and does not simulate neural elements (e.g., the spinal cord, nerve roots, or dura mater). Therefore, while the model provides critical insights into corrective forces and implant stresses, it cannot assess clinically important risks associated with multi-level releases, such as dural tears or nerve root injury. These specific clinical risks must be evaluated through future clinical trials or more sophisticated models incorporating neural tissue. Despite these limitations, this FEA provides valuable biomechanical insights into a complex clinical problem and lays the groundwork for future studies, which should include a larger cohort of virtual patients and more complex loading scenarios.

## 5 Conclusion

This finite element analysis provides a strong biomechanical rationale for a selective, rather than extensive, release strategy in the surgical correction of severe rigid AdIS. Our findings demonstrate that while adding PIVR to Ponte osteotomies is critical for enhancing deformity correction, there is an optimal extent of release. A targeted 4-level release centered on the apex yielded the best correction and lowest implant stress. A more extensive release proved less effective, a phenomenon explained by the loss of a stable “fulcrum” essential for efficient corrective force transmission. These results directly challenge the ‘more is better’ paradigm and provide a clear rationale for surgeons to prioritize an ‘optimal release’ strategy to improve outcomes while potentially reducing surgical morbidity. Future prospective clinical studies are warranted to validate these biomechanical insights.

## Data Availability

The raw data supporting the conclusions of this article will be made available by the authors, without undue reservation.
